# Supplementary material for: Observation and manipulation of quantum interference in a superconducting Kerr parametric oscillator
Source: Nat Commun. 2024 Jan 2;15:86. doi: 10.1038/s41467-023-44496-1 (PMC10762009; doi:10.1038/s41467-023-44496-1)
Supplement: Supplementary file 1 — Supplementary Information [file 41467_2023_44496_MOESM1_ESM.pdf]

Supplementary Information for "Observation and manipulation of quantum interference in a superconducting Kerr parametric oscillator"

1. DERIVATION OF THE HAMILTONIAN

In this section, we derive the Hamiltonian of a superconducting Kerr parametric oscillator (KPO) whose circuit diagram is shown in Supplementary Fig. 1. The Lagrangian of the circuit is given by

$$\mathcal{L} = \mathcal{T} - \mathcal{U},$$

where

$$\mathcal{T} = \left(\frac{\Phi_0}{2\pi}\right)^2 \frac{C_\mathrm{s} + (C_\mathrm{Ja} + C_\mathrm{Jb})/N}{2}\dot{\phi}^2 = \left(\frac{\Phi_0}{2\pi}\right)^2 \frac{C_\mathrm{K}}{2}\dot{\phi}^2,$$
$$\mathcal{U} = -NE_\mathrm{Ja}\cos\left(\frac{\phi}{N} - r_\mathrm{a}\varphi_\mathrm{ex}\right) - NE_\mathrm{Jb}\cos\left(\frac{\phi}{N} + r_\mathrm{b}\varphi_\mathrm{ex}\right).$$

Here, Φ_0 is the magnetic flux quantum, $C_\mathrm{K} \equiv C_\mathrm{s} + (C_\mathrm{Ja} + C_\mathrm{Jb})/N$, $\varphi_\mathrm{ex} \equiv 2\pi\Phi_\mathrm{ex}/\Phi_0$, and $r_\mathrm{a} + r_\mathrm{b} = 1$. Using the formula $A\cos(x) + B\sin(x) = R\cos(x - \lambda)$, where $R = \sqrt{A^2 + B^2}$ and $\tan(\lambda) = B/A$, we obtain

$$\mathcal{U} = -NE_\mathrm{J}\cos\left(\frac{\phi}{N} - \lambda\right),$$

where

$$E_\mathrm{J} = \sqrt{E_\mathrm{Ja}^2 + E_\mathrm{Jb}^2 + 2E_\mathrm{Ja}E_\mathrm{Jb}\cos(\varphi_\mathrm{ex})},$$
$$\lambda = \arctan\left(\frac{E_\mathrm{Ja}\sin(r_\mathrm{a}\varphi_\mathrm{ex}) - E_\mathrm{Jb}\sin(r_\mathrm{b}\varphi_\mathrm{ex})}{E_\mathrm{Ja}\cos(r_\mathrm{a}\varphi_\mathrm{ex}) + E_\mathrm{Jb}\cos(r_\mathrm{b}\varphi_\mathrm{ex})}\right).$$

To account for the flux bias modulation, we introduce a variable change, $\phi \to \phi + N\lambda$, and decompose φ_ex into static and oscillating parts, i.e., $\varphi_\mathrm{dc} + \varphi_\mathrm{ac}(t)$. The oscillating part is the parametric pump given by $\varphi_\mathrm{ac}(t) = 2\varphi_\mathrm{ac0}\cos(\omega_\mathrm{p}t)$. Since $\varphi_\mathrm{ac0} \ll 2\pi$, we take the Taylor expansion at $\varphi_\mathrm{ex} = \varphi_\mathrm{dc}$:

$$\mathcal{U} \approx -N(E_\mathrm{J0} + E_\mathrm{J1}\varphi_\mathrm{ac} + E_\mathrm{J2}\varphi_\mathrm{ac}^2)\cos\left(\frac{\phi}{N}\right),$$

where

$$E_\mathrm{J0} = \sqrt{E_\mathrm{Ja}^2 + E_\mathrm{Jb}^2 + 2E_\mathrm{Ja}E_\mathrm{Jb}\cos(\varphi_\mathrm{dc})},$$
$$E_\mathrm{J1} = -\frac{1}{E_\mathrm{J0}}E_\mathrm{Ja}E_\mathrm{Jb}\sin(\varphi_\mathrm{dc}),$$
$$E_\mathrm{J2} = -\frac{1}{2E_\mathrm{J0}^3}\left[E_\mathrm{Ja}E_\mathrm{Jb}(E_\mathrm{Ja}^2 + E_\mathrm{Jb}^2)\cos(\varphi_\mathrm{dc}) + (E_\mathrm{Ja}E_\mathrm{Jb})^2\left\{\cos^2(\varphi_\mathrm{dc}) + 1\right\}\right].$$

Here, the φ_ac term gives the parametric pump, whereas the φ_ac^2 term induces the AC Stark shift.

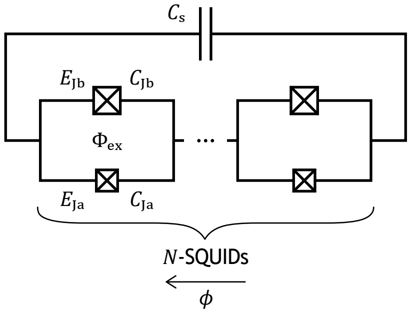

SUPPLEMENTARY FIG. 1. Circuit diagram of a superconducting KPO. The KPO consists of N direct current superconducting quantum interference devices (DC SQUIDs). E_Ja and C_Ja (E_Jb and C_Jb) represent the Josephson energy and the capacitance of the smaller (larger) junction in each DC SQUID, respectively. C_s denotes the shunting capacitance of the KPO, and Φ_ex is the external magnetic flux threaded through one DC SQUID.

The resulting Hamiltonian is (hereafter, we add a hat to an operator for clarity)

$$\hat{\mathcal{H}} = 4E_\mathrm{C}\hat{N}_\phi^2 - NE_\mathrm{K}\cos\left(\frac{\hat{\phi}}{N}\right) + 2\gamma\cos(\omega_\mathrm{p}t)\cos\left(\frac{\hat{\phi}}{N}\right),$$

where $\hat{N}_\phi$ is the conjugate number operator, $E_\mathrm{C} \equiv e^2/2C_\mathrm{K}$, $E_\mathrm{K} \equiv E_\mathrm{J0} + 2E_\mathrm{J2}\varphi_\mathrm{ac0}^2$, and $\gamma \equiv -NE_\mathrm{J1}\varphi_\mathrm{ac0}$. The φ_ac^2 term was averaged out by using $\langle\varphi_\mathrm{ac}^2\rangle = 2\varphi_\mathrm{ac0}^2$ and absorbed into E_K.

We move to the occupation-number representation by defining

$$\hat{N}_\phi = \mathrm{i}N_0^\phi(\hat{a}^\dagger - \hat{a}), \quad \hat{\phi} = \phi_0(\hat{a}^\dagger + \hat{a}),$$

where $\hat{a}$ and $\hat{a}^\dagger$ are the ladder operators for the KPO, and $N_0^\phi = \sqrt[4]{E_\mathrm{K}/32NE_\mathrm{C}}$ and $\phi_0 = \sqrt[4]{2NE_\mathrm{C}/E_\mathrm{K}}$ are the zero-point fluctuations. The full Hamiltonian, including a single-photon drive with frequency ω_d and amplitude β, is

$$\hat{\mathcal{H}} = \hbar\omega_\mathrm{K0}\hat{a}^\dagger\hat{a} - NE_\mathrm{K}\left[\cos\left(\frac{\hat{\phi}}{N}\right) + \frac{1}{2}\left(\frac{\hat{\phi}}{N}\right)^2\right] + 2\gamma\cos(\omega_\mathrm{p}t)\cos\left(\frac{\hat{\phi}}{N}\right) + 2\beta\cos(\omega_\mathrm{d}t + \phi_\mathrm{d})(\hat{a}^\dagger + \hat{a}),$$

where $\hbar\omega_\mathrm{K0} = \sqrt{8E_\mathrm{C}E_\mathrm{K}/N}$. After expanding the cosine terms, normal ordering, and rotating wave approximation in a frame rotating with $\omega_\mathrm{p}/2$, we obtain Eq. (1) in

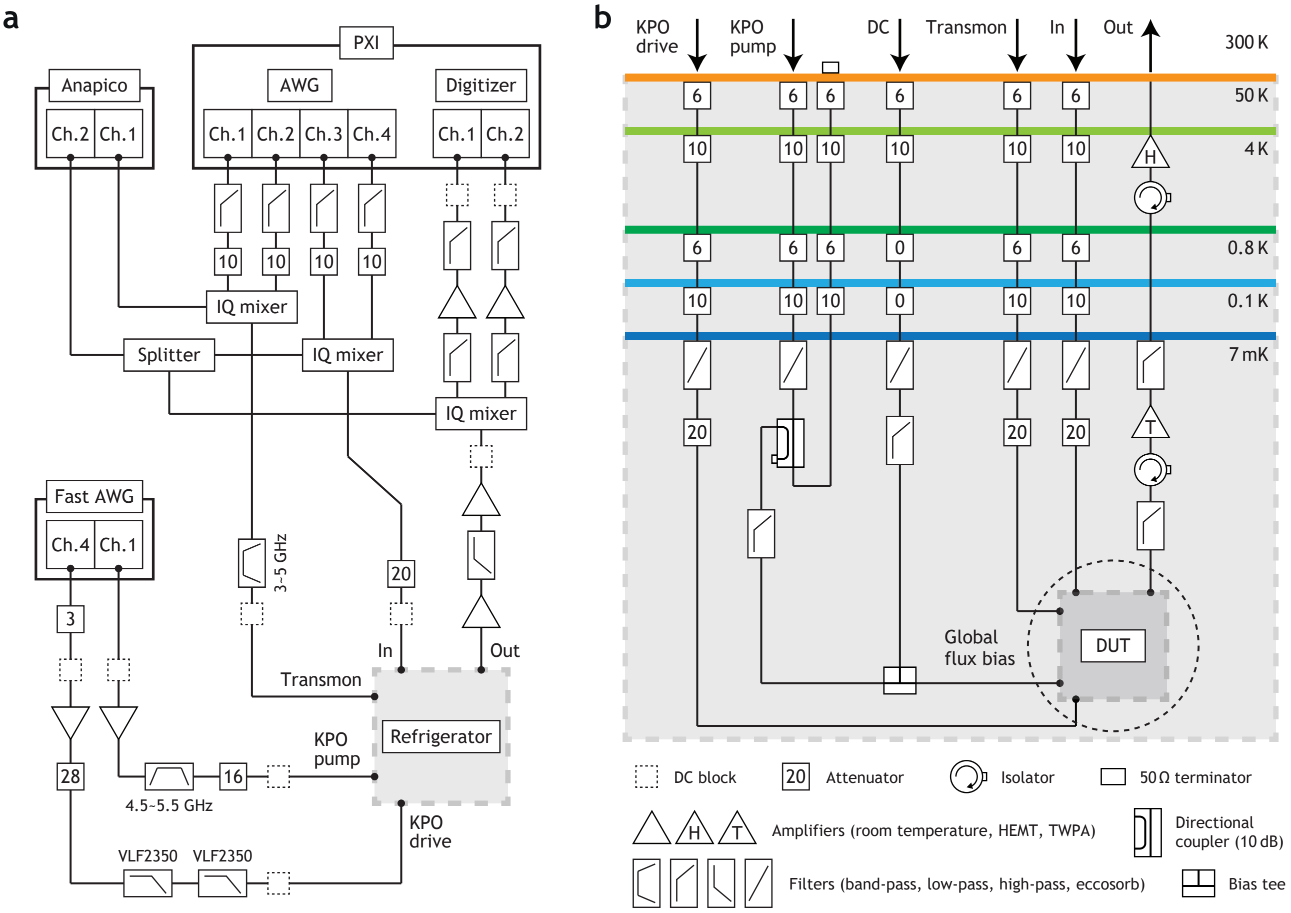

SUPPLEMENTARY FIG. 2. **a**,**b** Experimental configuration of room-temperature electronics (a) and cryogenic components (b). The 50-Ω-terminated port next to the pump line serves to dissipate unused pump power. The DC line is not used in this work. The isolator at the mixing chamber is a double-junction isolator. The cables for the traveling wave parametric amplifier (TWPA) pump are not shown for simplicity.

the main text:

$$\hat{\mathcal{H}}/\hbar = \Delta\hat{a}^\dagger\hat{a} - \frac{K}{2}\hat{a}^\dagger\hat{a}^\dagger\hat{a}\hat{a} + \frac{P}{2}(\hat{a}^\dagger\hat{a}^\dagger + \hat{a}\hat{a}) + \beta\left[\hat{a}^\dagger e^{-i(\Delta_d t+\phi_d)} + \hat{a}e^{+i(\Delta_d t+\phi_d)}\right]. \tag{1}$$

Here, $\Delta(\equiv \omega_K - \omega_p/2)$ is the KPO-pump detuning, where $\omega_K(\equiv \omega_{K0} - K)$ is the transition frequency between the $|0\rangle$ and $|1\rangle$ states, $\Delta_d(\equiv \omega_d - \omega_p/2)$ is the drive-pump detuning, $\hbar K(\equiv E_C/N^2)$ is the self-Kerr coefficient, and $\hbar P(\equiv \varphi_{ac0}E_{J1}\sqrt{2E_C/NE_K})$ is the amplitude of the pump.

2. ELECTRONICS AND CABLING

The pulses for the transmon and the readout resonator were generated by arbitrary waveform generators (AWGs), local oscillators, and IQ mixers. For the KPO, pulses were generated using a 65 GSa/s AWG (Keysight M8195A) with a bandwidth of 25 GHz because the fast and large bandwidth control of the KPO is crucial for this study. However, the actual bandwidth of these pulses is limited to about 2 GHz due to the low-pass filters used to protect the transmon from the strong displacement pulses. No notable pulse distortion was observed in our oscilloscope.

3. SYSTEM PARAMETERS

The measured system parameters are presented in Supplementary Table 1. It should be noted that the longitudinal relaxation time of the KPO T_1^K varied significantly over time. During the initial stage of the measurement, T_1^K exceeded 30 µs. However, after one month, it decreased to less than 10 µs and did not recover. For instance, the data shown in Supplementary Figs. 5 and 6d were obtained before the degradation of T_1^K, which was 34 µs at that time. As for other data in Supplementary Fig. 6, T_1^K was only 5 µs. These values are used for the simulations in Supplementary Figs. 5 and 6. The T_1^K and

SUPPLEMENTARY TABLE 1. Measured system parameters. Φ_0 is the magnetic flux quantum. "RR" stands for readout resonator. "PNS" stands for photon number splitting. "VPM" stands for vacuum state parity measurement. PNS and VPM data can be found in Supplementary Fig. 5. For more rigorous definitions, see Supplementary Eqs. (1) and (5).

Physical quantity	Symbol	Value	Method
Number of the DC SQUIDs in the KPO	N	10	
External magnetic flux threaded through one DC SQUID	Φ_{ex}	$0.25\,\Phi_0$	
Transition frequency between $\lvert 0\rangle$ and $\lvert 1\rangle$ states of the KPO	$\omega_{\mathrm{K}}/2\pi$	2.531 GHz	Rabi (Fig. 1)
Self-Kerr coefficient of the KPO	$K/2\pi$	2.86 MHz	Rabi (Fig. 1)
Longitudinal relaxation time of the KPO	T_1^{K}	8 µs	
Transverse relaxation time of the KPO	T_2^{K}	3 µs	Ramsey
Transition frequency between $\lvert \mathrm{g}\rangle$ and $\lvert \mathrm{e}\rangle$ states of the transmon	$\omega_{\mathrm{T}}/2\pi$	3.793 GHz	Rabi and Ramsey
Anharmonicity of the transmon	K_{T}/h	214 MHz	Two-tone spectroscopy
Longitudinal relaxation time of the transmon	T_1^{T}	52 µs	
Transverse relaxation time of the transmon	T_2^{T}	36 µs	Ramsey
Cross-Kerr coefficient between the KPO and the transmon	$\chi_{\mathrm{KT}}/2\pi$	1.63 MHz	PNS and VPM
Higher-order cross-Kerr coefficient	$\chi_{\mathrm{KKT}}/2\pi$	−9 kHz	VPM
Number of thermally excited photons in the KPO	n_{th}	0.10	PNS and VPM
Resonance frequency of the RR	$\omega_{\mathrm{R}}/2\pi$	7.614 GHz	
Cross-Kerr coefficient between the transmon and the RR	$\chi_{\mathrm{TR}}/2\pi$	0.4 MHz	

SUPPLEMENTARY TABLE 2. Parameters for the pulse shape. "σ" represents the standard deviation of Gaussian pulses. For flap-top Gaussian pulses, σ indicates the standard deviation of the pulse edges. For cat generation pulses, the definition of the ramping time τ_{ramp} can be found in Fig. 1b. The time interval between two $\pi/2$ transmon pulses for the parity measurement is 300 ns (Supplementary Fig. 5b).

Operation	Data	Element	Pulse shape	Pulse length (ns)	σ (ns)
Rabi	Fig. 1	KPO	Flat-top Gaussian	Variable	1
		Transmon	Gaussian (π)	2000	500
Photon number splitting	Supplementary Figs. 3d and 5a	KPO	Gaussian	10	2.5
		Transmon	Gaussian (π)	2000	500
State preparation	Fig. 2b ($\lvert 0\rangle + \lvert 1\rangle$)	KPO	Flat-top Gaussian	420	1
	Fig. 2b ($\lvert 1\rangle$)	KPO	Flat-top Gaussian	840	1
	Supplementary Fig. 6c	KPO	Gaussian	10	2.5
	Supplementary Fig. 6e	KPO	Gaussian	242.8	2.5
	Supplementary Fig. 6f	KPO	Gaussian	359.0	2.5
Cat generation	Figs. 2–4	KPO	$\sin^2(\pi t/2\tau_{\mathrm{ramp}})$	300	
Counterdiabatic	Figs. 2–4	KPO	$\sin(\pi t/\tau_{\mathrm{ramp}})$	300	
Cat Rabi	Fig. 4a	KPO	Flat-top Gaussian	Variable	1
X/2 gate for cat Ramsey	Fig. 4b	KPO	Flat-top Gaussian	84	1
X/2 gate for QPT	Fig. 4c	KPO	Flat-top Gaussian	43	1
Parity measurement	All tomographies	KPO	Gaussian	10	2.5
		Transmon	Gaussian ($\pi/2$)	25	6.25
Transmon readout	All data	Readout resonator	Flat-top Gaussian	2500	4

T_2^{K} values in Supplementary Table 1 were measured at the final stage of experiment, during which most of the data in the main text were collected.

4. RABI OSCILLATIONS

The Rabi oscillations induced by the drive with the frequency ω_{d} were simulated using the following Hamiltonian:

$$\hat{\mathcal{H}}_{\mathrm{Kd}}/\hbar = \Delta' \hat{a}^\dagger \hat{a} - \frac{K}{2}\hat{a}^\dagger\hat{a}^\dagger\hat{a}\hat{a} + \beta\big(\hat{a}^\dagger + \hat{a}\big)\,, \qquad (2)$$

where $\Delta'(\equiv \omega_{\mathrm{K}} - \omega_{\mathrm{d}})$ is the KPO-drive detuning. For the Rabi oscillations induced by the pump, Supplementary Eq. (1) with $\beta = 0$ were used.

The simulation data of Rabi oscillations in the KPO $|0\rangle$ state population are shown in Supplementary Fig. 3 with the experimental data already shown in Fig. 1c,d. By comparing the simulation and experimental data, we extract P, K, and $2\omega_{\mathrm{K}}$ (Supplementary Fig. 3c). Note that K diverges if P is greater than K. This limits the P/K ratio to 1.01.

The photon number splitting data are shown in Supplementary Fig. 3d. The distance between peaks is about 1.6 MHz. To detect the $|0\rangle$ state population selectively, the frequency marked by the arrow was chosen for the transmon π-pulse. Here, the length of the transmon π-pulse must be sufficiently long (2 µs in this work) to ensure that the bandwidth of the pulse is sufficiently narrower than the spacing between transmon peaks. (The details on the pulse shape can be found in Supplementary Table 2.)

5. WIGNER TOMOGRAPHY

A. Kerr correction

The Wigner function is the expectation value of the parity operator at the phase-space coordinate α [1]:

$$W(\alpha) = \frac{2}{\pi}\,\mathrm{Tr}\Big[\hat{D}^\dagger(\alpha)\rho\hat{D}(\alpha)\hat{P}\Big],$$

where $\hat{D}(\alpha) = \exp\big(\alpha\hat{a}^\dagger - \alpha^*\hat{a}\big)$ is the displacement operator, $\hat{P} = \exp\big(\mathrm{i}\pi\hat{a}^\dagger\hat{a}\big)$ is the photon number parity operator, and ρ is the density matrix. For a KPO, the experimental displacement operator is given by

$$\hat{D}_{\mathrm{K}} = \exp\left\{-\frac{\mathrm{i}}{\hbar}\int_0^{\tau_{\mathrm{d}}} dt' \left[\beta(t')(\hat{a}^\dagger + \hat{a}) - \frac{K}{2}\hat{a}^\dagger\hat{a}^\dagger\hat{a}\hat{a}\right]\right\}, \qquad (3)$$

where τ_{d} is the length of the displacement pulse. Here, the K term distorts the Wigner tomography. Thus, we must eliminate the evolution induced by this term. The difficulty is that the β and K terms do not commute. Our method relies on the assumption that the dynamics induced by these two terms are separable for a short time (the Baker–Campbell–Hausdorff formula). This suggests that once we obtained the density matrix of the KPO from the Wigner tomography and QST-CGAN, ρ_{CGAN}, we can reverse this unwanted Kerr evolution by using the time-independent correction operator $\hat{U}_{\mathrm{cor}}$ if the displacement pulse is short enough. Our approach is outlined in Supplementary Fig. 4a.

We introduce an ansatz for $\hat{U}_{\mathrm{cor}}$:

$$\hat{U}_{\mathrm{cor}} = \exp\left(-\frac{\mathrm{i}}{\hbar}\frac{Kt_{\mathrm{cor}}}{2}\hat{a}^\dagger\hat{a}^\dagger\hat{a}\hat{a}\right), \qquad (4)$$

where t_{cor} is the Kerr correction time. Note that the sign of the Kerr term in Supplementary Eq. (4) is opposite to that in Supplementary Eq. (3) to "reverse" the Kerr evolution. Then, we correct the unwanted Kerr evolution by multiplying $\hat{U}_{\mathrm{cor}}$ to ρ_{CGAN}, i.e., $\hat{U}_{\mathrm{cor}}\rho_{\mathrm{CGAN}}\hat{U}^\dagger_{\mathrm{cor}}$. We call this process the Kerr correction.

To determine the conditions under which the unwanted Kerr evolution is correctable, we calculate the fidelity between the target state and the reconstructed state obtained from QST-CGAN. We then study how the fidelity changes as a function of displacement pulse length. We choose three states, the even cat, coherent, and $|0\rangle + |5\rangle$ states. The sizes of the cat and coherent states α_{c} are both 1.6 such that the mean photon numbers of these three states are similar. The shape of the displacement pulses is Gaussian whose standard deviation is a quarter of the pulse length. As shown in Supplementary Fig. 4b, if the length of a Gaussian displacement pulse is less than $1/(20K)$, the fidelity after the Kerr correction is close to 1, indicating that the unwanted Kerr evolution is correctable.

We also simulate how the performance of the Kerr correction depends on the size of a cat state α_{c}. Supplementary Figure 4c shows that with our experimental configuration ($\tau_{\mathrm{d}} = 10$ ns and $K/2\pi \approx 3$ MHz), the faithful Kerr correction is possible up to $\alpha_{\mathrm{c}} = 1.6$. The optimal correction time is determined by fitting the data in Supplementary Fig. 4d with a linear function; for $\tau_{\mathrm{d}} = 10$ ns, we obtain $t_{\mathrm{cor}} = 3.8$ ns, which is used throughout this work.

B. Calibration

In order to perform Wigner tomography, it is necessary to determine the relationship between the AWG setting value and the resulting displacement in phase space. This calibration process is carried out in two steps: setting value (V) $\rightarrow$ displacement pulse amplitude (Hz) $\rightarrow$ phase-space coordinate. The first step, which involves the conversion from volt to hertz, is accomplished by fitting the parity measurement of the vacuum state and photon number splitting data (Supplementary Fig. 5a,b) using the following Hamiltonian in a frame rotating with

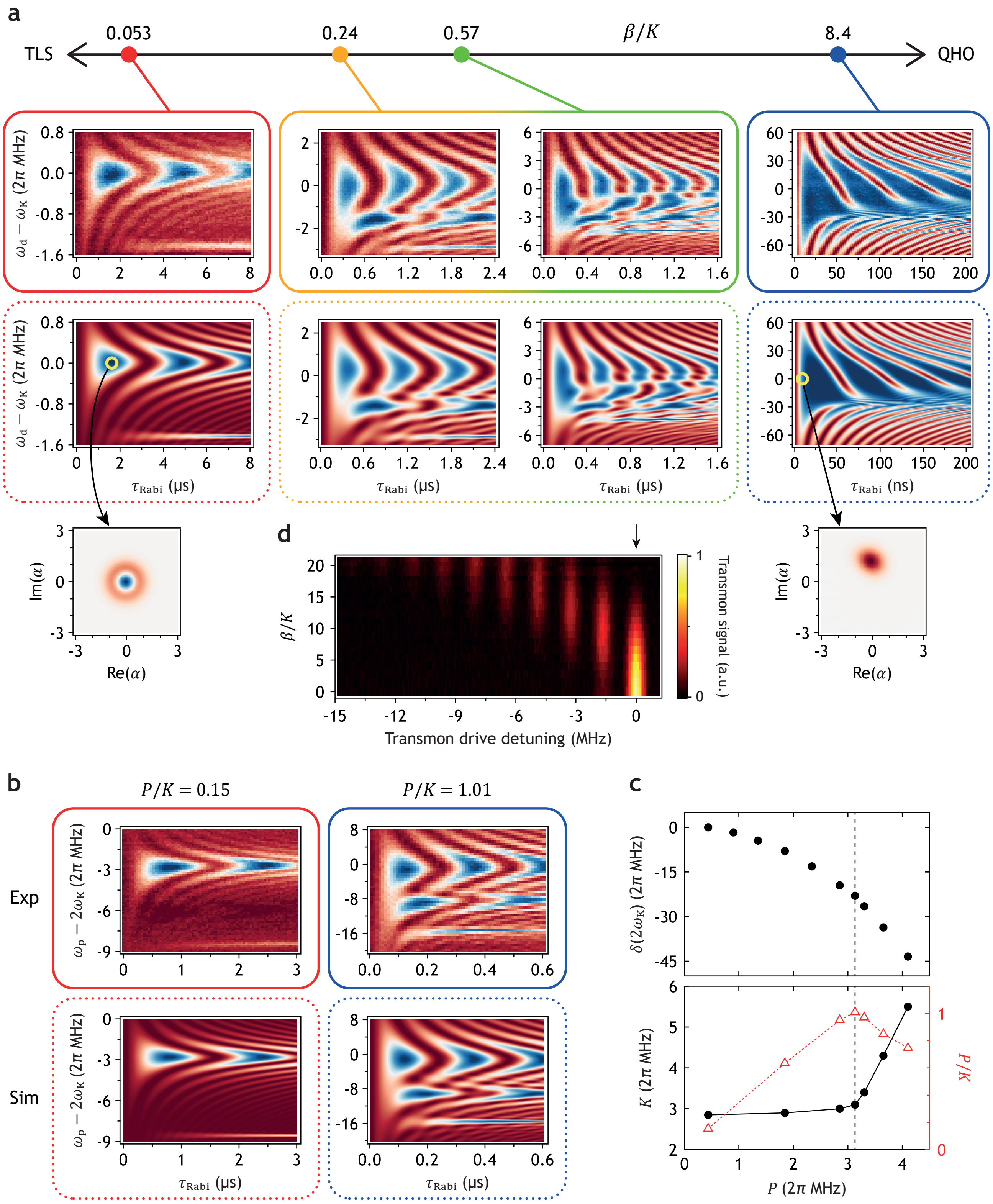

SUPPLEMENTARY FIG. 3. **a**,**b** Rabi oscillations in the $|0\rangle$ state population of the KPO induced by the drive (a) and pump (b). Plots in solid frames are the experimental data, and plots in dotted frames are the data from simulations without relaxations. Wigner tomographies in (a) show the states at the time indicated by yellow circles (simulation). **c** P dependence of the AC Stark shift $\delta(2\omega_{\mathrm{K}})$ and K. The AC Stark shift induced by the drive is negligibly small. The vertical dashed line indicates the pump strength used for this work. **d** Photon number splitting of the transmon spectrum induced by the non-zero photon number state of the KPO. The arrow indicates the transmon drive frequency used to detect the KPO Rabi oscillations.

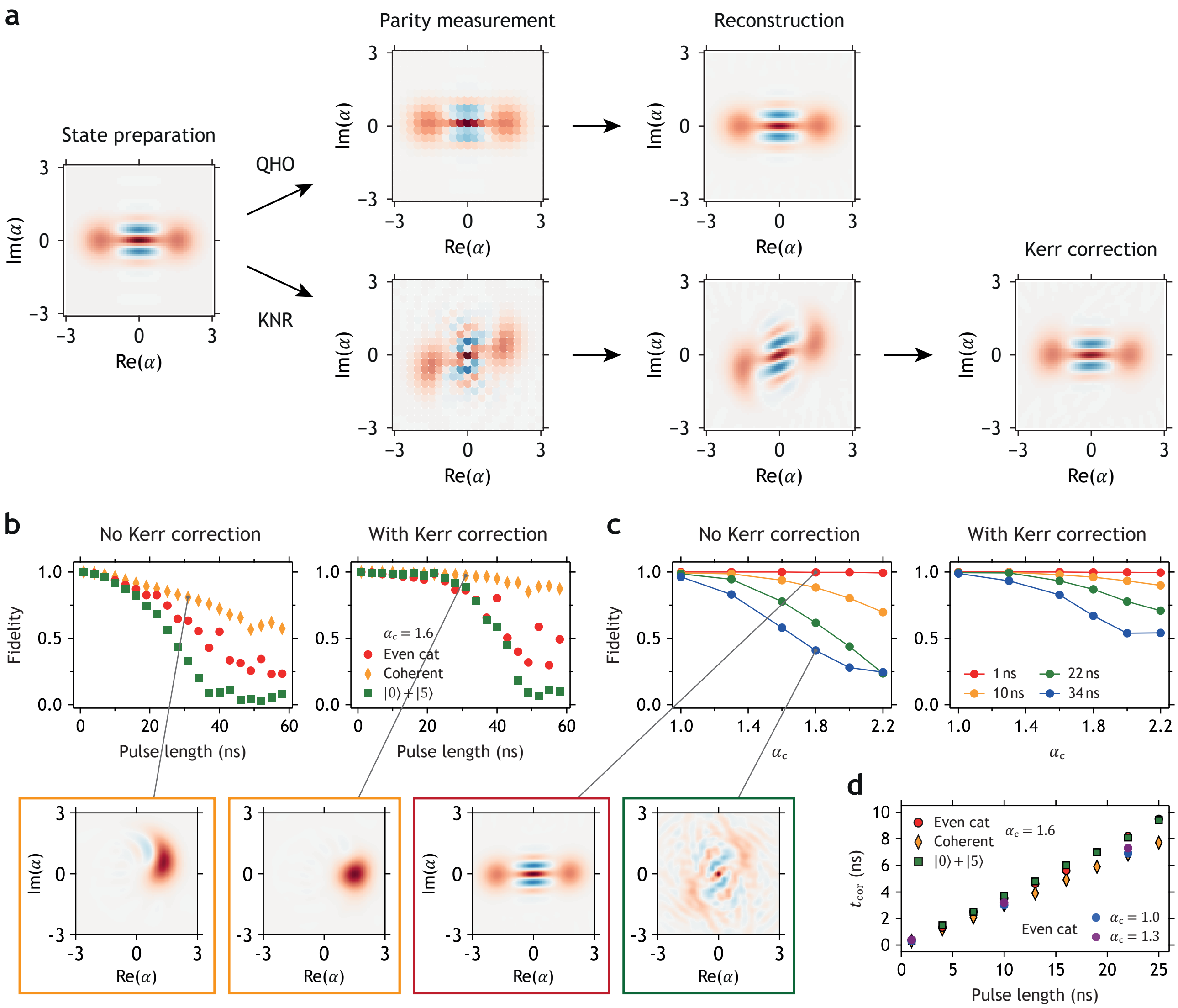

SUPPLEMENTARY FIG. 4. **a** Standard procedure for Wigner tomography of a QHO (upper row) and our approach for a Kerr nonlinear resonator (KNR) (lower row). "Reconstruction" refers to the Wigner plot of the density matrix extracted from the "Parity measurement" data using QST-CGAN. The original state is the even cat state with a size of $\alpha_c = 1.6$. **b**,**c** Fidelity between the original state and the reconstructed state as a function of displacement pulse length or α_c, both with and without Kerr correction. Examples of reconstructed Wigner tomographies are shown in colored rectangles. **d** Relationship between the pulse length and t_{cor} where the fidelity is maximized. All data and plots in this figure are simulation results without considering relaxation effects. The Kerr coefficient of the KPO is assumed to be 3.2 MHz.

the transmon drive frequency ω_{Td} [2]:

$$\begin{aligned}\hat{\mathcal{H}}_{\rm fit}/\hbar = {} & \Delta_{\rm K}\hat{a}^\dagger\hat{a} - \frac{K}{2}\hat{a}^\dagger\hat{a}^\dagger\hat{a}\hat{a} \\ & + \beta\left(\hat{a}^\dagger e^{-i\Delta_{\rm dT}t} + \hat{a}e^{+i\Delta_{\rm dT}t}\right) \\ & + \hbar\Delta_{\rm T}\hat{b}^\dagger\hat{b} - \frac{K_{\rm T}}{2}\hat{b}^\dagger\hat{b}^\dagger\hat{b}\hat{b} + \beta_{\rm T}(\hat{b}^\dagger + \hat{b}) \\ & + \chi_{\rm KT}\hat{a}^\dagger\hat{a}\hat{b}^\dagger\hat{b} + \chi_{\rm KKT}\hat{a}^\dagger\hat{a}(\hat{a}^\dagger\hat{a} - 1)\hat{b}^\dagger\hat{b},\end{aligned} \tag{5}$$

where $\Delta_{\rm K} \equiv \omega_{\rm K} - \omega_{\rm Td}$, $\Delta_{\rm dT} \equiv \omega_{\rm d} - \omega_{\rm Td}$, and $\Delta_{\rm T} \equiv \omega_{\rm T} - \omega_{\rm Td}$. The calibration results from both data sets are identical (inset of Supplementary Fig. 5a).

The second step involves comparing the Wigner function of the thermal state with the parity measurement data taken at a time interval that is half of the period (indicated by the vertical dashed line in Supplementary Fig. 5b). By doing this, we can determine the relationship between the displacement pulse amplitude and the phase-space coordinate (inset of Supplementary Fig. 5b) as well as the mean photon number of the thermal state. The mean photon number can also be extracted from photon number splitting data. The calibration from these measurements is consistent with that from the Rabi measurement (Supplementary Fig. 3a).

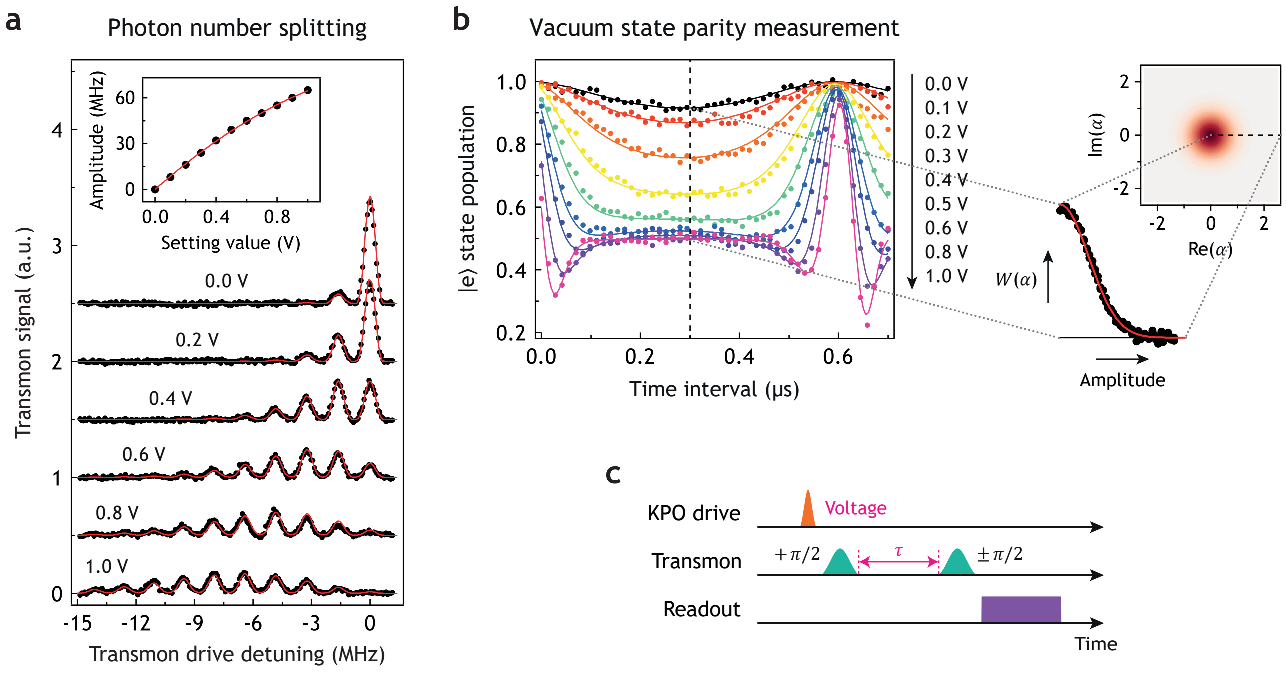

SUPPLEMENTARY FIG. 5. Displacement pulse amplitude calibration. **a** Calibration using photon number splitting. The data points were shifted by 0.5 steps for clarity. **b** Calibration using parity measurement of the vacuum state. All solid lines in (a) and (b) represent simulation results obtained using Supplementary Eq. (5). The inset in (a) shows the results of fitting by comparing the measurements and simulations in (a) and (b). The red solid line in the inset represents a simple quadratic fit. **c** Pulse sequence for (b). The control parameter is the time interval τ between two $\pi/2$ pulses. Further information regarding the pulse conditions can be found in Supplementary Table 2.

C. Sample tomographies

We take Wigner tomographies of four states, namely, the $|0\rangle+|1\rangle$ state, the coherent state, and two cat states, to confirm our measurement accuracy. We first choose the $|0\rangle+|1\rangle$ state because it is the simplest state that shows quantum coherence and does not suffer from the Kerr evolution during the state preparation. The state is prepared using a long and weak pulse whose condition is similar to that found in the rightmost plot of Supplementary Fig. 3a. (The details on the pulse shape can be found in Supplementary Table 2.) The measured Wigner tomography and its reconstruction are shown in Supplementary Fig. 6a. In addition to the tomography, we perform the photon number splitting measurement that shows the diagonal elements of the density matrix (Supplementary Fig. 6b). We also simulate the transmon signal by solving the Lindblad master equation with Supplementary Eq. (5) and the density matrix from the reconstruction as the initial KPO state (its diagonal part is shown in the inset).

Next, we prepare coherent and cat states (Supplementary Fig. 6c–f) by adjusting the length of the Gaussian pulse, while keeping the standard deviation as a constant ($\sigma = 2.5$ ns). When the pulse length is significantly larger than σ, the system evolves almost freely in the tail of the pulse with the period $T_{\mathrm{K}}(\equiv 1/K)$, which is 349 ns for our KPO when the pump is off. The three- and two-component cat states are generated by applying a Gaussian pulse with 116.4 ns ($= T_{\mathrm{K}}/3$) and 174.5 ns ($= T_{\mathrm{K}}/2$) tails, respectively [3, 4]. (The total pulse length is twice the length of the tail. See Supplementary Table 2 for details.)

The fidelities between the reconstructed density matrix and the results of simulation using the Lindblad master equation with Supplementary Eq. (5) are 0.987 for Supplementary Fig. 6a, 0.992 for (c), 0.942 for (e), and 0.935 for (f).

6. CAT RABI AND RAMSEY SIMULATIONS

A. Cat Rabi oscillations in a TLS

As mentioned in the main text, the cat Rabi oscillations can be reproduced in a TLS. For this, the Rabi Hamiltonian with *two* drive tones with opposite detuning must be used:

$$\hat{\mathcal{H}}_{\mathrm{Rd}}/\hbar = \frac{\Omega_{\mathrm{R}}}{4}\left(\mathrm{e}^{-\mathrm{i}\Delta_{\mathrm{dT}}t} + \mathrm{e}^{+\mathrm{i}\Delta_{\mathrm{dT}}t}\right)(\hat{\sigma}_+ + \hat{\sigma}_-). \tag{6}$$

Here, Ω_{R} is the Rabi frequency, $\Delta_{\mathrm{dT}} \equiv \omega_{\mathrm{d}} - \omega_{\mathrm{TLS}}$, and $\hat{\sigma}_\pm \equiv (\hat{\sigma}_x \pm \mathrm{i}\hat{\sigma}_y)/2$, where $\hat{\sigma}_x$ and $\hat{\sigma}_y$ are Pauli operators.

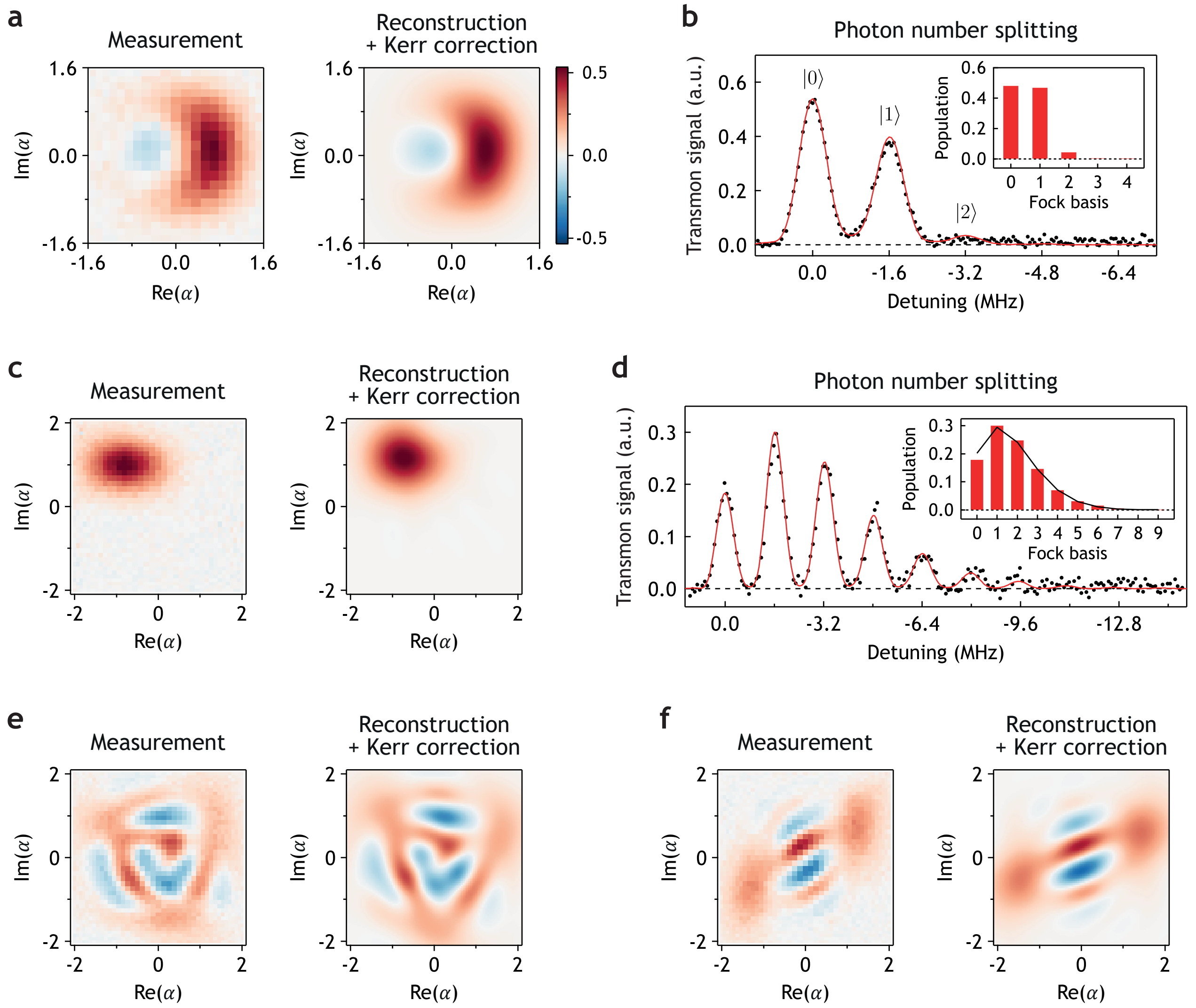

SUPPLEMENTARY FIG. 6. **a**,**b** Wigner tomography and photon number splitting of $|0\rangle + |1\rangle$ state. In (b), the black dots are experimental data; the red solid line is the simulation result using the density matrix obtained using QST-CGAN and the Lindblad master equation. The diagonal part of this density matrix (population of each Fock state) is shown in the inset. Note that the transmon spectrum is displayed from high to low frequencies for clarity. **c**,**d** Wigner tomography and photon number splitting of a coherent state. In the inset of (d), the black solid line shows the diagonal part of the density matrix obtained from the numerical simulation. **e**,**f** Three- and two-component cat states.

The typical Rabi Hamiltonian for a TLS is

$$\hat{\mathcal{H}}_{\mathrm{Rs}}/\hbar = \frac{\Omega_{\mathrm{R}}}{2}\left(\hat{\sigma}_{+}\mathrm{e}^{+\mathrm{i}\Delta_{\mathrm{dT}}t} + \hat{\sigma}_{-}\mathrm{e}^{-\mathrm{i}\Delta_{\mathrm{dT}}t}\right). \tag{7}$$

The Rabi oscillations simulated using Eqs. (6) and (7) are shown in Supplementary Fig. 7b.

B. Background ripples in cat Ramsey fringes

Supplementary Figure 7c,d shows the experimental and simulated cat Ramsey fringes. Our simulation reproduces not only the Ramsey pattern but also the background ripples, suggesting that these background ripples are not experimental artifacts. Since the number parity is not necessarily identical to the population of the $|{+}\mathrm{Cat}\rangle$ state, we also simulate this quantity and observe similar background ripples. As shown in Supplementary Fig. 7e, under the Z gate condition where the ripples are relatively weak (denoted by the circle), the Wigner tomographies before and after the second X/2 pulse are close to our expectation. However, under the condition with strong ripples (triangle), the states after the Z gate appear distorted.

The diamond in Supplementary Fig. 7c,d highlights a condition where the parity and population exhibit significant differences due to strong modulation of the pump frequency within a short time. This operation leads to a sudden change in $\Delta\hat{a}^{\dagger}\hat{a}$, resulting in population leakage

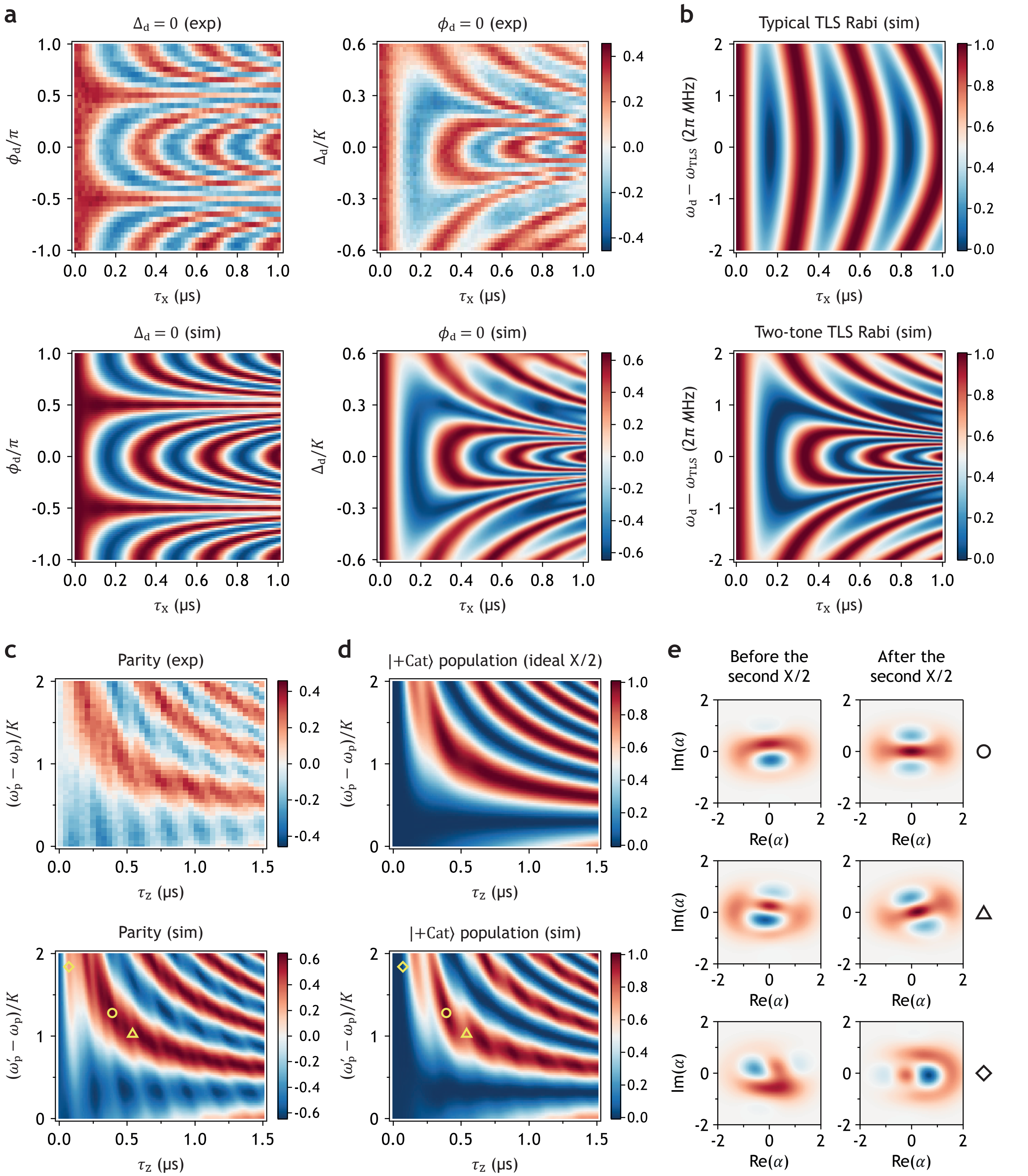

SUPPLEMENTARY FIG. 7. **a** Cat Rabi oscillations. The upper plots are experimental data, whereas the lower plots are simulations with $\Delta/2\pi = 1.0$ MHz and $\beta/2\pi = 0.65$ MHz. The colors represent the value of the Wigner function at $\alpha = 0$, i.e., the parity of the KPO. **b** Simulations of Fock state Rabi oscillation in a TLS using Supplementary Eq. (7) (upper plot) and Supplementary Eq. (6) (lower plot). $\Omega_R/2\pi = 3.0$ MHz. **c**,**d** Cat Ramsey fringes in the parity of the KPO and the population of the $|+\text{Cat}\rangle$ state. The upper plot of (c) is experimental data, whereas others in (c) and (d) are simulation data with $\Delta/2\pi = 0.5$ MHz. **e** Wigner tomographies of three selected conditions. Relaxations are not considered in all simulations.

from the qubit space. Our simulation confirms that the qubit space population is indeed only 0.436 under this condition. This type of error is an inherent property of the Z gate based on frequency modulation and cannot be resolved with an ideal X/2 gate. The upper plot of Supplementary Fig. 7d illustrates the simulated cat Ramsey with the ideal X/2 gate, which can be written in the operator form as

$$\hat{X/2} = |{-}\mathrm{iCat}\rangle\langle{+}\mathrm{Cat}| - \mathrm{i}\,|{+}\mathrm{iCat}\rangle\langle{-}\mathrm{Cat}|\,.$$

Note that when the frequency modulation is strong and the gate time is short, the cat Ramsey pattern remains imperfect, even though the background ripples are completely eliminated.

SUPPLEMENTARY REFERENCES

[1] A. Royer, *Wigner function as the expectation value of a parity operator*, Phys. Rev. A **15**, 449–450 (1977).

[2] A. Essig, Q. Ficheux, T. Peronnin, N. Cottet, R. Lescanne, A. Sarlette, P. Rouchon, Z. Leghtas, and B. Huard, *Multiplexed Photon Number Measurement*, Phys. Rev. X **11**, 031045 (2021).

[3] B. Yurke and D. Stoler, *The dynamic generation of Schrödinger cats and their detection*, Physica B+C **151**, 298–301 (1988).

[4] A. Miranowicz, R. Tanaś, and S. Kielich, *Generation of discrete superpositions of coherent states in the anharmonic oscillator model*, Quantum Opt. **2**, 253–265 (1990).
