# Supplementary material for: Observation and manipulation of quantum interference in a superconducting Kerr parametric oscillator
Source: Nat Commun. 2024 Jan 2;15:86. doi: 10.1038/s41467-023-44496-1 (PMC10762009; doi:10.1038/s41467-023-44496-1)
Supplement: Supplementary file 3 — Description of Additional Supplementary Files [file 41467_2023_44496_MOESM3_ESM.docx]

**Description of Additional Supplementary Files**

**Supplementary Movie 1: Tunnelling between two classical energy minima in phase space.** The movie was constructed based on the Wigner tomographies for Fig. 3b from 0.4 µs to 10.0 µs with a 0.2 µs step.
